# Locomotor Ataxy Caused by Train Movement

**Published:** 1873-04-01

**Authors:** G. C. Lawrence

**Affiliations:** Resident Physician, Hot Springs, Arkansas


### LOCOMOTOR ATAXY CAUSED BY TRAIN MOVEMENT.

BY DR. G. C. LAWRENCE, RESIDENT PHYSICIAN, HOT SPRINGS, ARKANSAS.

Among the many chronic ills that are brought to our notice for treatment, at the Hot Springs of Arkansas, we find, annually, an increasing number of invalids who are afflicted with nervous ataxia.

In effort to determine more fully the causation of this disease, the history, occupation, habits of life, hereditary predisposition, age, duration of the attack, progress of the malady—in fact, everything that it is deemed desirable to learn concerning each particular case—is properly investigated. The review of our practice for the past ten years satisfies us that a certain *occupation* is a source of the progressive ill, and that such *employment* is promotive of *locomotor ataxy.*

Our attention was first directed to this subject by the fact that so large a proportion of our patients were railroad employes—conductors, engineers, government mail agents, express messengers, route agents, baggage masters, brakemen, firemen, and others who travel much by rail.

We are convinced, by careful observation, that constant railroad traveling is abusive to health, and an injury to the brain, nervous, and muscular system. That such travel is always a struggle with the nerve and muscular system, in effort to keep up a due equilibrium of forces, to antagonize the bi-lateral curve, or zigzag movement of the engine and cars, called *train movement.*

It is doubtless the train movement that causes visual or perceptive derangement, and provokes the disturbances in the powers of co-ordination.

It is the complex-lateral-vibratory action, an impulse acting from the extremities and periphery to the center, that the system has to contend with in dynamic agency.

Train motion is mostly from side to side. When standing, it keeps up in the body a constant resisting-force—an attitude of fixed muscular action on the part of the traveler, as if ever in readiness for every action caused by the forces of motion.

The train movement demands constant vigilance in position, for counter-action. It is a constant tension, or strain, on a part of the muscular system ; consequently the brain and motor nerves must suffer.

*Train motion* is unlike ship movement. The action of motion upon the brain, nervous and muscular systems, on shipboard, or on steamboats, is different from that of a railroad train. Aboard ship, especially at sea, we have, generally, the long, undulating, propelling, or plunging motion of the vessel; on steamships or steamboats, at sea, and inland navigation, it is similar, and does not provoke the result of train motion.

The officers of the respective navies—English, French, and American—enjoy average health, and (for the dangers of the occupation) they generally reach a fair period of life. Locomotor ataxy, according to our experience, is rarely found—at least it is less marked—among sea-faring men, officers, sailors, and marines.

This subject merits further investigation. Railroad men, in these days of commercial prosperity, comprise a large class of the community in every city on the continent.

The liability to this fearful disease should be better understood, and a longer period of rest given to all traveling agents belonging to railroad companies. Those who run great distances on trains, always, more or less, feel the wear and tear upon the machinery of life. Those who run on fast trains, especially when doing night service, grow weary, and require more quiet sleep. On *straight roads* the fatigue and exhaustion in travel is less to the system, than on those that are *curved.* Accommodation trains (all slow trains) provoke less injury than the rapid movement of express trains.

If any medical gentleman—one who is accustomed to study the anatomy of the expression in health and disease—will scan the face of an engineer of a passenger train—watch his anxious look and his attitude when the train is moving, with one hand on reverse-bar and the other on the throttle; with a nervous vigilance watching every movement of the machinery of the locomotive; also, an ear alive to and quickened in sense ; educated to every sound, front and rear—he will surely feel satisfied that the duties of his place do not tend to longevity.

Give railroad employes more sleep, less physical fatigue, and it will doubtless serve to give health.

